# Rosemary Reduces Heat Stress by Inducing CRYAB and HSP70 Expression in Broiler Chickens

**DOI:** 10.1155/2018/7014126

**Published:** 2018-10-23

**Authors:** Shu Tang, Bin Yin, Jiao Xu, Endong Bao

**Affiliations:** College of Veterinary Medicine, Nanjing Agricultural University, Nanjing 210095, China

## Abstract

Heat stress negatively affects poultry production and animal health. In response, animals invoke a heat stress response by inducing heat shock proteins (HSPs). Scientists are actively seeking natural products that can enhance the heat shock response. The present study aimed at assessing the effects of a purified rosemary extract comprising antioxidant compounds on the heat shock response and HSP expression profile in broiler chickens. The response of broilers to HS in the presence of purified rosemary extract was assessed using an in vivo myocardial cell model. Pathological lesions of heart tissue were examined microscopically. The levels and activities of enzymes associated with heart damage and oxidative damage were detected. Immunohistochemical staining was performed for HSPs in myocardial cells. The results showed that lactate dehydrogenase (LDH), creatine kinase (CK), and myocardial CK (CKMB) levels were reduced by the purified rosemary extract before and during heat stress. Heat stress alone increased CK and CKMB levels. The levels of oxidative damage-associated enzymes were compared between the rosemary + heat stress and heat stress-alone groups. The results indicated that in terms of these enzymes, the purified rosemary extract induced a more antioxidative state. Pathological examinations showed that heat stress caused myocardial fiber fracture, karyopyknosis, and degeneration. The addition of purified rosemary extract ameliorated these lesions to some degree, preserving more of the basic structure. Heat stress decreased the cellular levels of crystallin alpha B (CRYAB) and HSP70. The addition of the purified rosemary extract significantly increased the levels of CRYAB and HSP70 during heat stress (*p* < 0.0001). Immunohistochemistry showed that after rosemary treatment, CRYAB and HSP70 showed more intense staining compared with the no heat stress control group. In the rosemary + heat group, after 10 hours of heat stress, the staining intensity of these two proteins remained higher than in the heat stress group. Thus, purified rosemary extract could induce high levels of HSP70 and CRYAB in chicken hearts before and during heat stress. Purified rosemary extract could be used to alleviate heat stress in broiler chickens.

## 1. Introduction

Heat stress (HS) affects animal production worldwide and has a significant impact on animal well-being. Animals experiencing HS tend to reduce their heat production by limiting feed intake, with subsequent negative effects on growth performance [1]. Stressful conditions, such as environmental, pathological, and nutritional disturbances, can generate a state of tension that evokes behavioral and physiological chain reactions that generally impair poultry performance. Therefore, HS has been a great concern among scientists and poultry producers for many decades, particularly in arid and tropical regions of the world, as well as in other climates because of surges in temperature during the spring and summer months [2, 3]. It is believed that heat-related pathologies, such as heat stroke, will soon become one of the most serious causes of mortality. Climatic shifts and other contemporary anthropogenic causes contribute to the increased prevalence of hyperthermia and associated death. During heat stroke, the core body temperature exceeds 40°C, which elicits acute tissue injury and multiorgan failure that is often fatal. Chicken (*Gallus gallus*) heart cells undergo necrosis after 5 hours of heat stress in vivo and in vitro [3, 4]. The involvement of HS as an inducer of oxidative stress has been acknowledged [5, 6]. Oxidative stress is defined as the presence of reactive species in excess of the available antioxidant capacity of animal cells [7, 8]. Many radicals and metabolic substances are potentially toxic and are defined as “reactive oxygen/nitrogen/chlorine species”[4, 9]. These substances are highly reactive and can modify several biologically cellular macromolecules, such as proteins, lipids, and nucleic acids (DNA and RNA) [10, 11]. Heat stress is one of the most challenging environmental conditions that affect commercial poultry. Thus, substantial attention has been paid to the role of nutritional additives to minimize the effects of HS [12, 13]. In our previous studies, we fed broiler chickens natural substances, such as coenzyme Q10 (Q10) and vitamin C, during heat stress. The chickens showed a reduced heat shock phenomenon in vivo, and their primary myocardial cells showed less damage compared with those of the HS-only group. In addition, indicators of oxidative stress, such as malondialdehyde (MDA), superoxide dismutase (SOD), and lactate dehydrogenase (LDH), were decreased after Q10 and vitamin C treatment [10, 14]. Therefore, we focused on identifying further natural substances that can ease HS of broilers in vivo.

The leaves of the plant *Rosmarinus officinalis* L. (rosemary) are commonly used as a spice, flavoring agent, and as a naturally occurring antioxidant. In the 1950s, it was reported that an extract of rosemary leaves contained high antioxidant activity. Subsequently, extracts from rosemary leaves were used commercially for their antioxidant activity [15–17]. Extracts of rosemary leaves have been used to prevent oxidation and to inhibit the oxidation of both animal fats and vegetable oils [18]. The antioxidant activity of rosemary leaves' extract is comparable to that of butylated hydroxytoluene (BHT) and butylated hydroxyanisole (BHA), which are synthetic antioxidants [19]. Rosemary leaves contain antioxidant compounds such as carnosol, carnosic acid, rosmaridiphenol, rosmanol, isorosmanol, epirosmanol, rosmariquinone, and rosmarinic acid. Our purified rosemary extract contains mainly carnosic acid and carnosol. Using an emulsification technique, carnosic acid can be made water soluble, making it easy to use in the clinic. Carnosic acid is a natural benzenediol abietane diterpene, with the formula C_20_H_28_O_4_. Carnosic acid belongs to the terpenoids, the largest class (over 50,000) of plant secondary metabolites, also known as isoprenoids or terpenes [16]. Carnosic acid contains two phenolic hydroxyl groups; therefore, it is often classified among the polyphenols. Carnosic acid (1) plus carnosol (2) have been suggested to account for over 90% of the antioxidant properties of rosemary extract [20], although this has not been systematically verified. In the present study, we aimed to investigate whether a purified rosemary extract could ease heat stress damage in broiler chickens.

The induction of heat shock proteins (HSPs) when animals are under several stresses is termed the heat shock response (HSR). The HSR plays a key role in cellular defense systems, in which HSPs act as protein chaperones to facilitate protein folding and the removal of aberrant proteins [21]. These properties contribute to the enhanced cellular survival produced following preconditioning stimuli, in which a subthreshold stimulus is used to raise endogenous HSP levels before the main stimulus [22]. When cells are under stress, several HSPs are induced, such as HSP70, HSP90, and HSP27. HSPs participate in several signaling pathways, including the oxidative stress pathway [23]. HSP70 is an important member of the HSP family and is highly conserved. Its cytoprotective function has been widely investigated; HSP70 senses oxidative damage and repairs unfolded or misfolded proteins. Furthermore, HSP70 can bind to and inhibit apoptotic proteins to regulate apoptosis under various environmental stresses. Our previous work indicated that HSP70 is significantly induced in chicken myocardial cells under heat stress and is associated with HSP70's protective function [24–26].

Small heat shock proteins (SHSPs) are ubiquitous components of protein quality control cell networks. As with other chaperones, SHSPs have a high capacity to bind unfolded proteins and facilitate substrate refolding [27, 28]. HSP27 and *α*B-crystallin belong to the SHSP superfamily and are functional, stress-induced SHSPs that are expressed in numerous tissues types, notably in muscles. The rapid upregulation of SHSPs is regulated by transcriptional and translational mechanisms [29]. HSP27 and *α*B-crystallin possess a homologous *α*-crystallin domain, which prevents actin microfilament disruption under stress conditions [30]. The effects of SHSPs on the cytoskeleton may be important in individual cell tolerance to stress through cytoskeletal stabilization [31].

To investigate whether purified rosemary extracts can ameliorate heat stress in chickens and whether rosemary could trigger HSP expression, we established a chicken in vivo heat stress model, followed by different durations of HS. The HS model allowed us to investigate the time course of rosemary's role in the myocardium and the associated HSP expression profiles.

## 2. Materials and Methods

### 2.1. In Vivo Myocardial Cell Model Establishment

The establishment of the in vivo HS model was described in detail in our previous report [5]. Briefly, one-day-old specific pathogen-free chickens were purchased from Qian Yuan Hao Biotechnology Company, Nanjing, China. The entire chicken population was vaccinated against Newcastle disease and infectious bursal disease on the 7th and the 14th days, respectively. The birds were given 7 days to acclimate to their new housing and to recover from environmental stress. One hundred chickens were randomly divided into two groups, designated as the HS group and the rosemary + HS group, respectively. Chickens in the rosemary + HS group were fed 3% rosemary nanoemulsion liquid (water soluble, kindly donated by the College of Life Sciences, Tsinghua University, Beijing, China), at a final concentration of 40 mg per day and given with water orally 7 days (280 mg) before HS. Chickens were allowed to drink water freely; normally, drug-added water would be drank and finished within 5 hours, then normal water would be given (room temperature: 25 ± 2°C). When HS was triggered, chickens in the HS and rosemary + heat groups were transferred to a preheated air chamber (GJ = 1, Suzhou Fengshi Laboratory Animal Equipment Co. Ltd, China) at 42 ± 1°C with 60–70% humidity for different durations of heat stress (0, 1, 3, 5, and 10 h). Chickens had free access to food and water during HS. All experiments were performed in accordance with the guidelines of the Animal Ethics Committee of Jiangsu Province (China) and were approved by the Institutional Animal Care and Use Committee of Nanjing Agricultural University, China.

#### 2.1.1. Purified Rosemary Extract Composition and Nanoemulsion Preparation

Purified rosemary extract was provided by Hainan Super Biotech Co. Ltd. Its composition was detected using high-performance liquid chromatography (HPLC). The chromatographic conditions are as follows: chromatographic column: Diamonsil® C18 (250 mm ∗ 4.6 mm, particle size 5 *μ*m); flow rate: 1.0 mL/min, equal degree elution; column temperature 35°C; sample quantity: 10 *μ*L; detection wavelength: 220 nm; and flow phase: acetonitrile: water: phosphoric acid (volume ratio) = 60 : 40 : 0.2. For the nanoemulsion, 1 g of purified rosemary extract (containing mainly carnosic acid > 95%, Hainan Super Biotech Co. Ltd., Lot. yp17070201) and 1.5 g of soy lecithin (Cargill Texturizing Solutions Deutschland GmbH & Co. KG, Germany) were dissolved in 15 g of caprylic/capric triglyceride (CCTG, Croda Co. Ltd., UK) at 60°C, and the carnosic acid lipid phase was added to a 82.5 g glycerol aqueous solution with 10% (*w*/*w*) glycerol at 60°C and emulsified by stirring at 1500 rpm for 1 min. Afterwards, the resulting preemulsion was homogenized via hot high-pressure homogenization (HPH, NS1001L, Niro Soavi, Italy) at 60°C for 4 cycles at 1000 bar. Finally, the resulting dispersion was cooled under ambient conditions to room temperature to obtain the carnosic acid-loaded nanoemulsion.

#### 2.1.2. Purified Rosemary Extract Nanoemulsion Stability Test

The purified rosemary extract nanoemulsion stability test was detected by HPLC according to the same method above.

### 2.2. Pathological Lesion of Heart Tissue In Vivo

Chicken heart samples of the different treatment groups (HS, rosemary + HS) were fixed with 4% methanol and then paraffin-embedded. The samples were then cut into 4 *μ*m serial sections and stained with hematoxylin (3 min) and eosin (1 min). The slices were then examined using light microscopy (ZEISS, Imager A2).

### 2.3. Detection of Heart Damage- and Oxidative Damage-Associated Enzymes

Collected samples were used to detect heart damage- and oxidative damage-associated enzymes; the creatine kinase (CK) (JC-1324), myocardial CK (CKMB) (JC-1301), LDH (JC-112), total- (T-) SOD (A001-1), total allene-oxide cyclase (T-AOC) (A012-5), and MDA (A003-2) activities in serum or heart samples were measured using commercial kits according to the manufacturer's instructions (Jiancheng, Nanjing).

### 2.4. Detection of HSP Expression

Total protein samples were extracted from chicken heart following HS using radioimmunoprecipitation assay lysis buffer with phenylmethylsulfonyl fluoride, and the protein concentration was determined using a bicinchoninic acid assay kit supplied by Life Technologies (Life Technologies; MK164230), according to manufacturer's instructions. Samples were denatured at 98°C for 15 min, and the concentration of HSP70, HSP27, and crystallin alpha B (CRYAB) was detected using a commercial ELISA kit (H264, Jiancheng, Nanjing, China, CSB-E12879C; CSB-EL006008CH, Cusabio Technology LLC, Houston, TX).

### 2.5. Immunohistochemical Staining (IHC)

When a heart sample was taken from a chicken corpse, 1 × 1 cm sample was immediately fixed into 4% paraformaldehyde and then paraffin-embedded. Dewaxed heart tissue sections (4 *μ*m) were fixed with HCl solution for antigen retrieval (2N HCL in distilled water, pH 0.6–0.9) for 20 min at room temperature (RT). After washing with PBS three times, endogenous peroxidase activity was inactivated by incubation in 3% (*v*/*v*) H_2_O_2_ for 10 min at RT. Subsequently, the sections were blocked with 5% bovine serum albumin (BSA) for 30 min at 37°C and then incubated with HSP primary antibodies (CRYAB: ADI-SPA-222-F, Enzo Life Science, USA; HSP27: ab26942, Abcam, USA; HSP70: ab53496, Abcam, USA) at 1 : 100 dilution for 2 h at 37°C. The negative controls were incubated with 1% BSA. After washing with PBS containing 1% Tween-20 three times, sections were incubated with a horseradish peroxidase goat anti-mouse (H + L) secondary antibody at 1 : 500 dilution for 1 h at 37°C. The sections were washed with PBS containing 1% Tween-20 three times and then treated with two drops of ready-made 3,3′-diaminobenzidine (DAB) substrate chromogen solution for 15 min until the desired brown color appeared. The sections were counterstained with hematoxylin and observed under a light microscope (Axio Imager A2, Zeiss, Jena, Germany).

### 2.6. Statistical Analysis

The software Curve Expert 1.3 was used to generate standard curves for the ELISAs. Data were compared with the baseline level (0 h in the HS group) by one-way ANOVA followed by Fisher's least significant difference (LSD) test using the SPSS (version 21 for Windows) and GraphPad Prism 6.0 software. Significant differences were indicated by *p* < 0.05, and highly significant differences were indicated by *p* < 0.01 in this study. All raw data presented were expressed as the mean ± standard deviation (SD). All experiments were repeated three times.

## 3. Results

### 3.1. HPLC Detection of Purified Rosemary Extract

**Figure 1:**
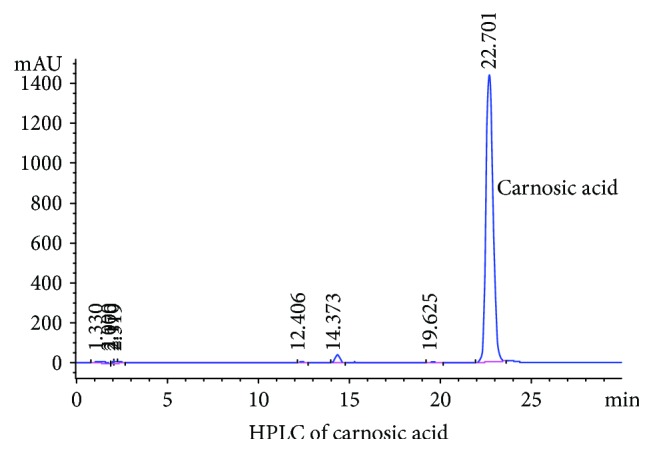
HPLC detection of purified rosemary extract.

According to the HPLC analysis, the main component of the rosemary eluate is carnosic acid (Figure 1). Therefore, after nanoemulsion formation, the main active ingredient is carnosic acid.

### 3.2. Purified Rosemary Extract Nanoemulsion Stability

Purified rosemary extract nanoemulsion stability results was provided by the manufacturer. According to the result, this nanoemulsion was stable (degradation rate < 5%) under normal condition during 12 months (Table 1). And when samples were put in 100°C for 12 hours, carnosic acid was stable. In our experiment condition, nanoemulsion was stable during 7 days. However, according to the previous report [32], carnosic acid was sensitive to light. So it needs to be stored in a light-blocking container.

### 3.3. Levels of Heart Damage-Associated Enzymes during Heat Stress

**Figure 2:**
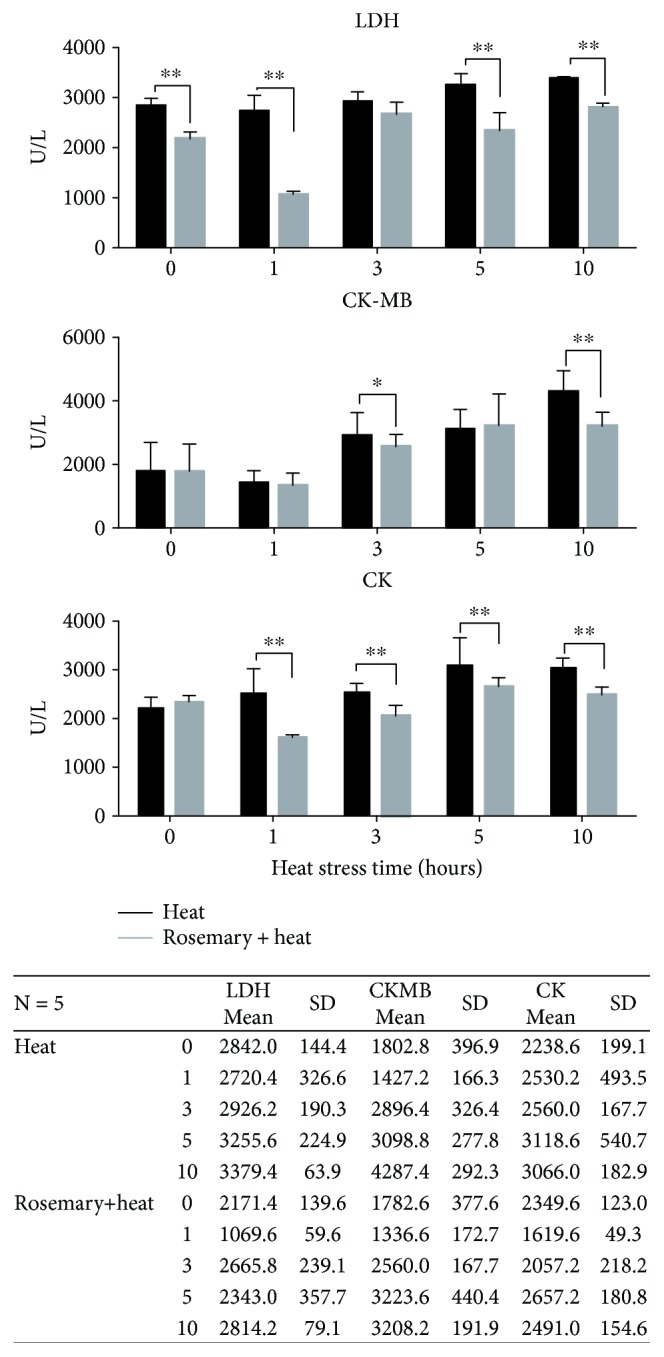
Cardiac damage-related enzymes detected in serum (^∗∗^*p* < 0.01, ^∗^*p* < 0.05). In the rosemary group, the lactate dehydrogenase (LDH) level remained lower than that in the heat stress (HS) group during 10 h of HS. Lower amounts of myocardial creatine kinase (CKMB) were present in the rosemary + heat than the HS group at 3 and 10 h. Creatine kinase (CK) levels were also in lower levels in the rosemary-treated group from 1 to 10 h of HS.

After heat stress, the levels of LDH, CK, and CKMB were detected in all experimental groups (Figure 2). According to our results, the LDH level in the serum was decreased after purified rosemary extract feeding for 7 days and was significantly (*p* < 0.01) lower than in the nonextract-treated group before heat stress. When exposed to heat stress, LDH in the rosemary + HS group remained lower than that in the HS group, especially at 1 h (about three times lower than HS group), 5 h, and 10 hours. The CK and CKMB levels showed similar patterns to LDH. After HS, the CK and CKMB levels were significantly higher than those at 0 h of HS. The levels in the rosemary groups were lower than those in the HS group, especially at 1 h of HS.

### 3.4. Levels of Oxidative Damage-Associated Enzymes in Serum during Heat Stress

**Figure 3:**
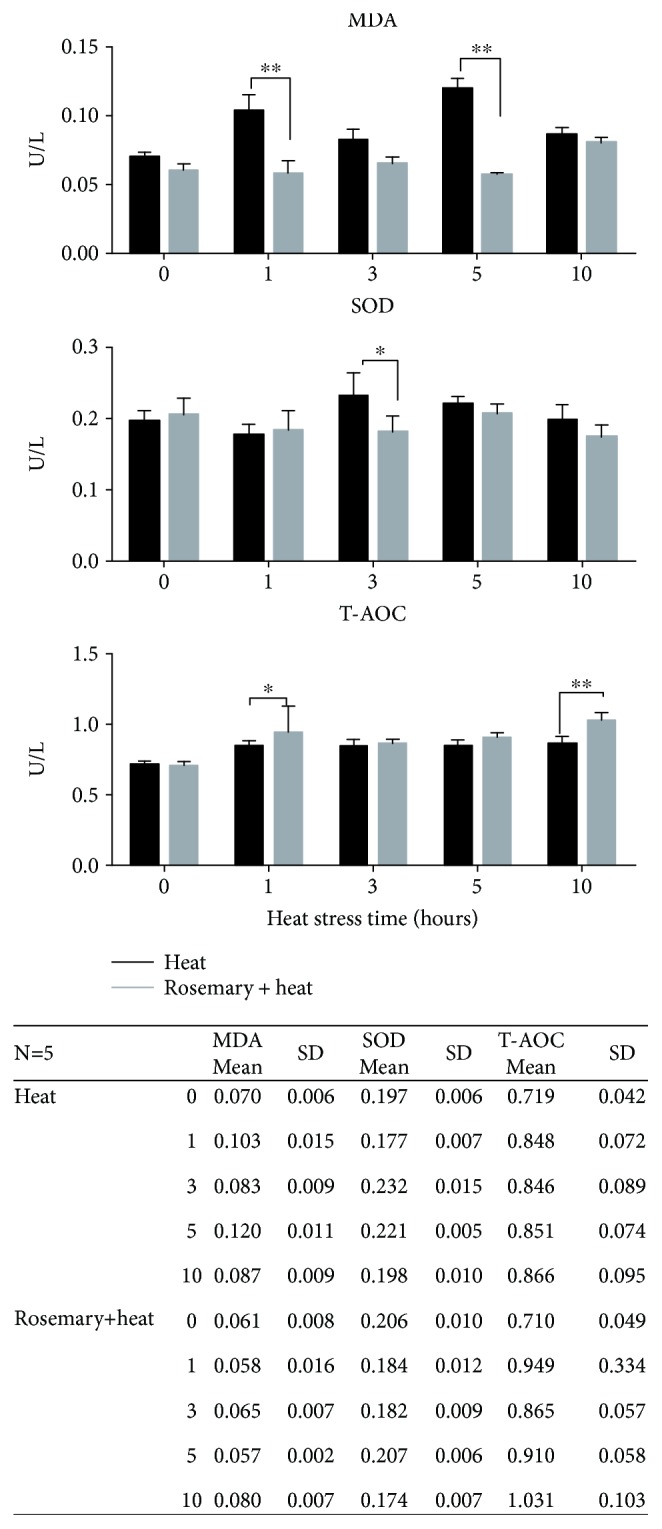
Oxidation-related enzymes detected in serum (^∗∗^*p* < 0.01, ^∗^*p* < 0.05). Malondialdehyde (MDA) levels were significantly lower at 3 and 5 h after heat stress in the rosemary group. Superoxide dismutase (SOD) showed a lower level only at 3 h in the rosemary group. Total allene-oxide cyclase (T-AOC) showed higher levels at 1 and 10 h in the rosemary group compared with that in the HS group.

Levels of T-SOD, T-AOC, and MDA in serum (Figure 3) were detected after heat stress. The results showed that in the HS group, when the chickens suffered from heat stress, the levels of MDA increased significantly from 1 h to 10 h, especially at 1 h and 5 h (*p* < 0.01). The rosemary group showed similar levels of T-SOD, T-AOC, and MDA compared with those in the control group, which were significantly lower than those in the HS group (*p* < 0.01, especially at 1 and 5 h). Pretreatment with purified rosemary extract before heat stress significantly increased the level of T-AOC at 1 h (*P* < 0.05) and 10 h (*p* < 0.01) compared with that in the HS-only group. At 3 h, the rosemary-treated group presented lower levels of SOD compared with those in the HS group (*p* < 0.05).

### 3.5. Pathological Examination of Chicken Heart during Heat Stress

**Figure 4:**
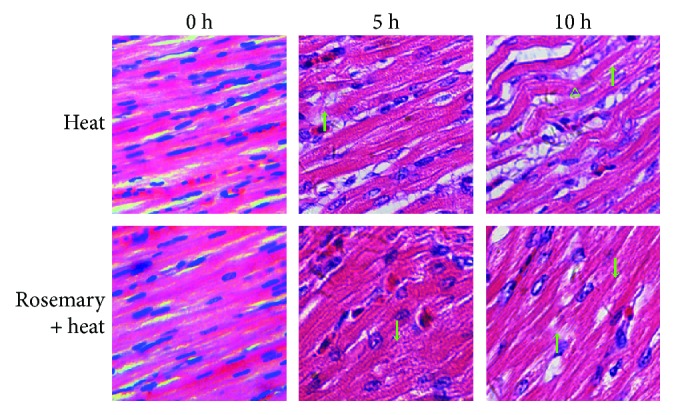
Pathological changes in different treatment groups (hematoxylin and eosin staining, magnification 400x, 1 bar = 10 *μ*m). No pathological changes were observed under normal conditions. When chickens suffered 5 h of heat stress, myocardial fiber fractures (↑) were observed in the heat stress (HS) group. Lesions in the rosemary + heat group showed acute degeneration (↓). At 10 h of heat treatment, karyopyknosis (△) and degeneration (↓) were observed in the HS group, whereas the hearts in the rosemary + heat group retained their basic structure, although fiber fracture (↑) and acute degeneration (↓) can be observed.

Pathological indications of injury were observed after heat stress in the HS and rosemary + heat groups (Figure 4). No pathological changes were observed under normal conditions. When chickens suffered 5 h of heat stress, myocardial fiber fractures were observed in the HS group; whereas the lesions in the rosemary + heat group were relatively less severe at this stage. When the duration of heat stress reached 10 h, karyopyknosis and degeneration were observed in the HS group; however, the hearts in the rosemary + heat group still retained their basic structure, although karyopyknosis and hemorrhage between cardiac muscle fibers were observed.

### 3.6. HSPs Expression during Heat Stress and Rosemary Treatment

**Figure 5:**
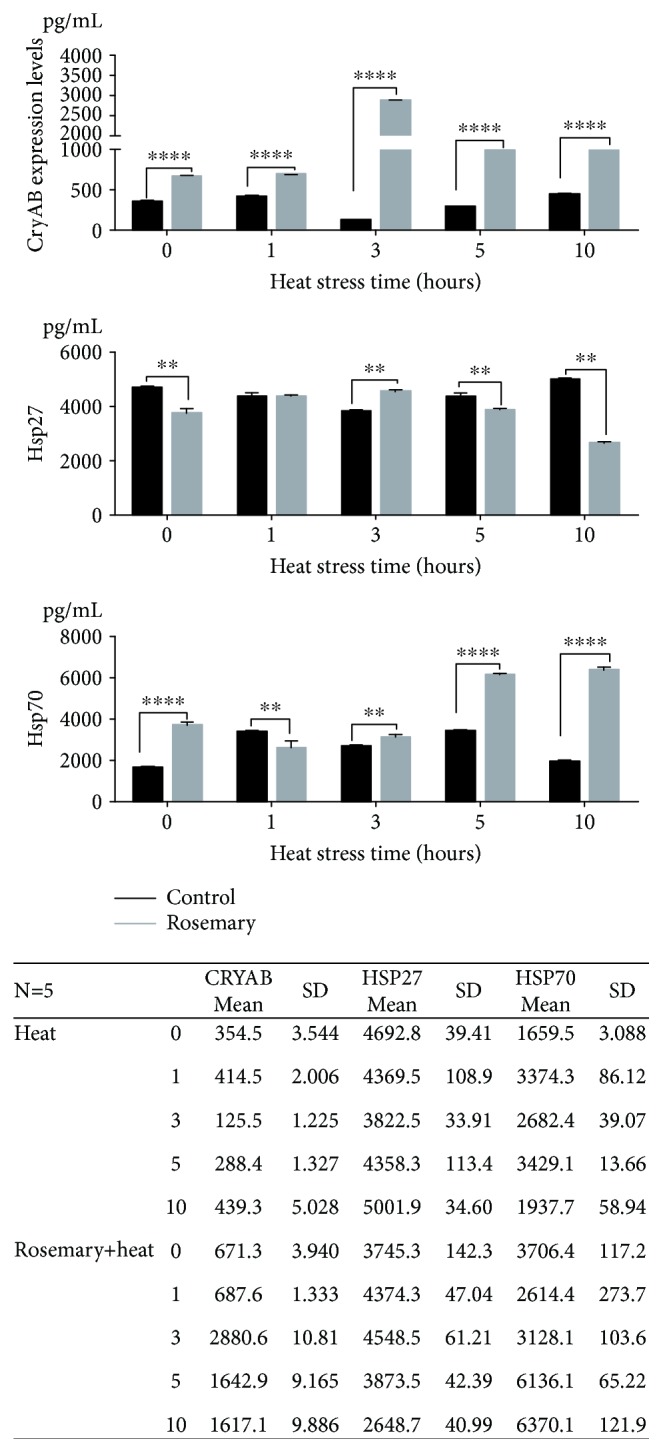
Heat shock protein (HSP) expression in different treatment groups (^∗∗∗∗^*p* < 0.0001, ^∗∗^*p* < 0.01).

HSP expression results are shown in Figure 5. The results showed that levels of CRYAB in chicken hearts increased significantly after rosemary treatment at 0 h of heat stress. In the HS group, CRYAB levels decreased at 3 and 5 h. However, by contrast, the CRYAB levels increased significantly in the rosemary + heat group from 3–10 h (*p* < 0.0001) compared with those in the HS group. HSP70 showed higher levels from 5–10 h in the rosemary + heat group (*p* < 0.0001). Before heat stress, HSP70 levels were also much higher in the no treatment group (0 h, *p* < 0.0001). At 1 h of HS, the HSP70 level was lower but then increased thereafter. HSP27 showed the opposite trend. Before heat stress, the rosemary group showed lower levels than the control, which increased at 3 h, then decreased compared with those in the HS group (*p* < 0.01).

### 3.7. Localization and Density of HSPs in Chicken Heart

**Figure 6:**
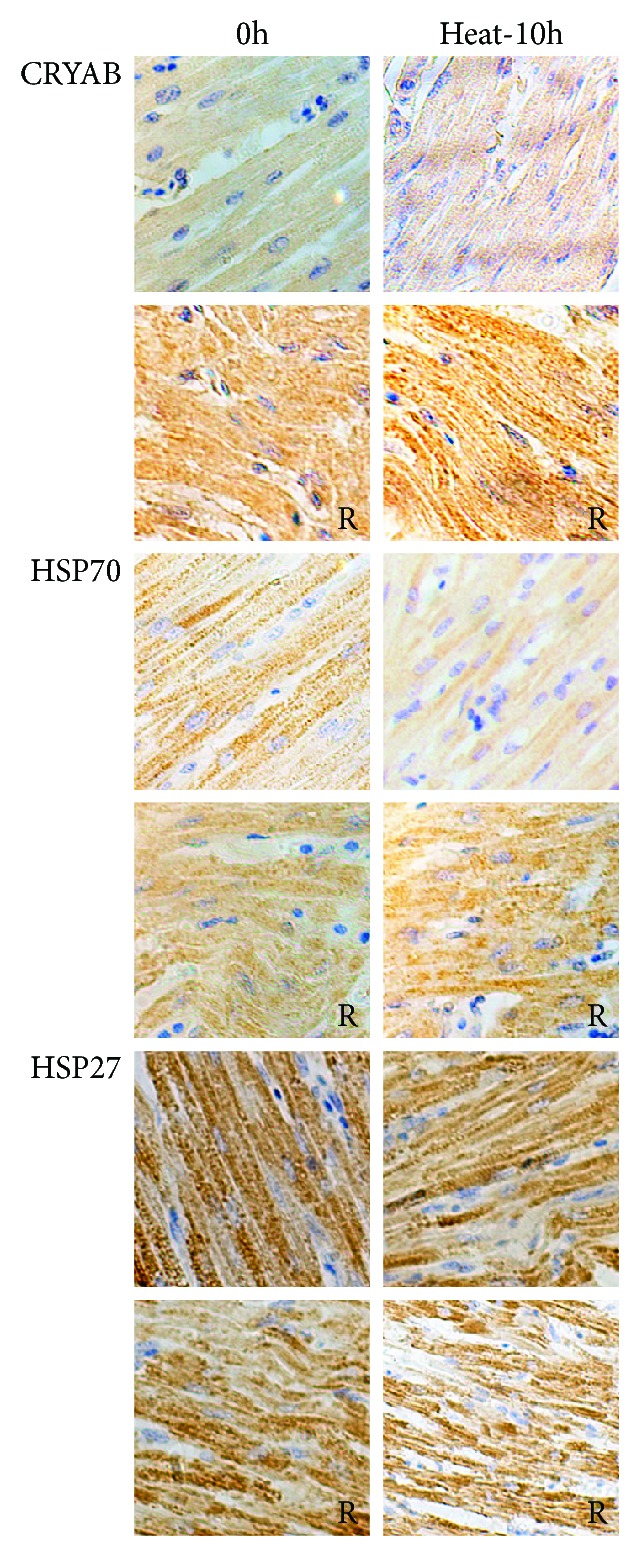
Immunohistochemical analysis of heat shock proteins (HSPs) in chicken heart tissue (1 bar = 10 *μ*m). After rosemary treatment, crystallin alpha B (CRYAB) and HSP70 showed higher staining intensity in the no heat stress (HS) group and in the 10 h heat stress group. However, HSP27 showed higher staining intensity before and after HS, except in the 10 h rosemary + heat group, in which the staining intensity was lower than in the control.

The immunohistochemistry results were consistent with the ELISA results (Figure 6). After rosemary treatment, CRYAB and HSP70 staining intensities were higher before heat stress compared with those in the nontreated control group. In the rosemary + heat group after 10 h of heat stress, the staining intensities of CRYAB and HSP70 were higher than those in the HS group. HSP27 showed a high staining intensity in chicken heart before and after treatment; however, in the rosemary + heat at 10 hours, the HSP27 staining intensity was decreased compared with that of the HS group.

## 4. Discussion

Heat stress is a type of nonspecific stressor that affects livestock's welfare and even contributes to death. In our previous studies, we confirmed that after a short period of heat stress (1 h), the enzymes related to heart damage, such as LDH, CK, and CKMB, could be detected in the blood serum and supernatant of rat myocardial cells. Our previous in vivo and in vitro research in rats confirmed that the cause of heat-induced sudden death was heart cell damage (cell necrosis and cell degeneration) [5, 33]. The heart is the most important organ in animals and humans, and previous clinical reports of human cases demonstrated that thermal tolerance to heat stress is impaired in patients with cardiovascular diseases [34, 35]. Research suggested that carnosic acid has a strong cardioprotective effect against isoproterenol-induced myocardial injury in mice [36]. In the present study, LDH, CK, and CKMB levels in the rosemary + HS group were lower than those in the HS group, which indicated less cell damage [5, 14]. Carnosic acid bioavailability was shown by Doolaege et al. [37] and Jordán et al. [38], who reported that carnosic acid was assimilated in muscular tissue and remained in the circulatory system for several hours [39]. Pathological lesions were more severe, with damage characterized by necrosis, in the HS group during heat stress, whereas the rosemary + heat group showed fewer pathological changes (acute degeneration) compared with those in the HS group.

In animal breeding, heat stress can be alleviated by two ways, physically, such as by spraying water in the aviary or chemically (feeding with antistress substances, such as vitamin C or glutamine). These substances function mainly in an antioxidative manner. HS activates several apoptosis pathways, especially those associated with oxidative stress [4, 8]. Rosemary (*Rosmarinus officinalis*; Lamiaceae) is widely used commercially, not only as a culinary herb but also as an antioxidant in foods, nutritional supplements, and cosmetics [15]. Carnosic acid, the major polyphenolic diterpene of rosemary plant, has been shown to possess antimicrobial, neuroprotective, hepatoprotective, antiobesity, anti-inflammatory, anticancer, and antidepressant properties [20]. Purified rosemary extract has also been recognized as a healthy food component by the European Food Safety Authority (EFSA) [20]. Upon oxidation, carnosic acid displays antioxidative capacities that depend upon the lipid composition of the matrix and more so upon the oxidative conditions [9, 40]. MDA, SOD, and T-AOC are indicators of oxidative stress in animals [14]. Our results showed that the rosemary group had lower MDA levels at 1 and 5 h compared with those in the HS group. Lower SOD levels were observed at 3 h, and higher T-AOC levels were observed at 1 and 10 hours in the rosemary group. The antioxidative effect of carnosic acid has been confirmed by the European Commission and assigned the number E392 (Commission Directives 2010/67/EU and 2010/69/EU repealed in 2013 by EU regulation 231/2012 and 1333/2008) (classified “antioxidant: extracts of rosemary”). According to the EU regulation, only deodorized purified rosemary extracts containing carnosic acid and carnosol are considered as additives. Indeed, carnosic acid and its derivative carnosol are listed as key antioxidant compounds in purified rosemary extracts, and E392 dosage limitations are expressed as levels of carnosic acid and carnosol, rather than of the whole rosemary extract. Application areas are food matrices, including oils, animal fats, sauces, bakery wares, meat, and fish products. Chinese food additive regulation GB2760-2011 approves the use of rosemary extracts under the Chinese numbering system CNS 04.017. The antioxidative activities of carnosic acid have been widely confirmed, and our results partly confirmed these activities in terms of the levels of several oxidative stress-related enzymes in chickens under heat stress. In a future study, we will examine the associated antioxidative signaling pathways in cell lines in vitro.

CRYAB (HSPB5) is a member of the SHSP family (HSPB1–HSPB10) that acts as a chaperone and is located in several organs. CRYAB is the main chaperone of the cytoskeleton. In heart cells, the cytoskeleton plays a key role in maintaining the physical shape of cells [30, 41]. The cytoskeleton could avoid the endoplasmic reticulum stress (ER stress), contributing to protecting the function of mitochondria in vivo and in vitro. Heart cells contain 60% of the total mitochondria, which provide ATP and oxygen to the whole body. Our previous research in mammalian (rat) cells in vivo and in vitro showed CRYAB expression and localization in heart cells, which confirmed that CRYAB might play the important role in heart cells [33]. In the present study, the CRYAB level was significantly increased after rosemary treatment, by about 2-fold compared with that in the 0 h HS group. After heat stress, the rosemary +h eat group showed a more than 10-fold increase in CRYAB levels compared with that in the HS group, and the level remained higher until 10 h. This suggested that rosemary pretreatment might induce CRYAB overexpression before and during HS. In the HS group, CRYAB levels decreased at 3 and 5 h, which was consistent with our previous study in which CRYAB was overused during acute heat stress, in which it may participate in binding with F-actin or other cytoskeletal components in myocardial cells [42]. The immunohistochemistry results also confirmed that CRYAB was expressed at high levels in the rosemary group.

HSP70 is an important and highly conserved member of the HSP family. Its cytoprotective function has been widely investigated; it senses oxidative damage and repairs unfolded or misfolded proteins. HSP70 can bind to and inhibit apoptotic proteins to regulate apoptosis under various environmental stresses. Our previous study indicated that HSP70 is significantly induced in chicken myocardial cells under HS [43]. In the present study, rosemary pretreatment also induced HSP70 expression, by over 2-fold compared with that in the control group after 5 h of HS, which was significantly higher than that in the HS group. These results suggested rosemary can preinduce HSP70 expression before stress occurs, and when under acute stress, HSP70 levels decreased at 1 and 3 h in the rosemary + heat group, which suggested that there was sufficient HSP70 in myocardial cells under heat stress. However, by 5 h of HS, the level of HSP70 increased significantly, probably in response to the more severe cell damage. By contrast, HSP70 levels were increased from in 1–5 h in the HS group, but then decreased at 10 h, accompanied by severe damage. This confirmed that HSP70 could exert anticell damage and apoptosis functions [44]. The immunohistochemistry result confirmed that in the rosemary + heat group, HSP70 was present at a much higher level than that in the control group, which was consistent with the ELISA results.

HSP27 belongs to the SHSP superfamily and is a functional, stress-induced SHSP that is expressed in numerous tissues types, notably in muscles [29]. HS causes extensive cytoskeletal and mitochondrial damage, as well as uncoupling of oxidative phosphorylation. HSP27 binds to numerous nonnative proteins via an oligomeric complex and is thus one of the most efficient chaperones in terms of quantity of substrate binding [27]. In this study, rosemary treatment did not induce increased HSP27 levels before HS, and HSP27 levels only increased at 3 h in the rosemary + heat group and then decreased. However, in the HS group, the HSP27 level was decreased at 3 h but then increased from 5–10 h of exposure to HS, which was a different pattern to that of CRYAB and HSP70. According to the immunohistochemistry results, HSP27 was present at a higher level in normal conditions compared with that of CRYAB and HSP70, which might explain why the HSP27 level did not change dramatically during HS. According to previous research, HSP27 has two phosphorylation sites at Ser78 and Ser82 [45]. Phosphorylation of HSP27 has been suggested to play an important role in the regulation of F-actin dynamics in response to stress [46]. The microfilament network is one of the earliest targets of oxidative stress, and phosphorylation of HSP27 is strongly induced by reactive oxygen metabolites; therefore, a previous study investigated the role of HSP27 phosphorylation in regulating actin dynamics in response to oxidative stress [27, 28]. In the present study, HSP27 levels decreased at 10 h in the rosemary + heat group, and it may be phosphorylated to play a protective role in chicken myocardial cells. However, antibodies that target the different phosphorylated forms of chicken HSP27, but not the nonphosphorylated form, are not easy to find. The role, if any, of HSP27 in HS-induced myocardial damage in chickens will be confirmed in further in vitro studies.

We also used Q10 and vitamin C to feed chicken in the past study, to compare their anti-heat stress activities. Q10 is a substance that is necessary for the heart, which can mainly induce HSP70 expression in chicken heart cell [10], and vitamin C can also induce higher CRYAB expression [14]. According to our observation during heat stress, the Q10, Vc, and rosemary groups all showed much better behavior compared to HS group when chickens were subjected to different durations of heat stress. However, it is likely that these different substances have different effects through different signaling pathways. These aspects require further research. However, the results of the present study suggest that combinations of different natural substances would have improved anti-heat stress effects.

In conclusion, purified rosemary extract can induce high levels of HSP70 and CRYAB in chicken heart in normal and heat stress conditions. It showed a protective effect for the chicken heart when under heat stress. Future studies should investigate the signal pathways involved using cell models.

## Figures and Tables

**Table 1:** Stability test of purified rosemary extract nanoemulsion during 12 months.

Date	Months	Indicators		Degradation rate (compared to 0 month)
		Character	Content of carnosic acid	
14 April 2016	0	Light yellow brown emulsion, slightly thick	102.09%	0
14 July 2016	3	Light yellow brown emulsion, slightly thick	101.63%	0.45%
15 Oct. 2016	6	Light yellow brown emulsion, slightly thick	100.81%	1.25%
13 Jan. 2017	9	Light yellow brown emulsion, slightly thick	99.85%	2.19%
17 April 2017	12	Light yellow brown emulsion, slightly thick	97.53%	4.47%

Condition: temperature: 30°C ± 2°C; humidity: 45% ± 5%

## Data Availability

The data used to support the findings of this study are available from the corresponding author upon request.
